# Impact of Point-of-Care Ultrasound on the Management of Abdominal Pain in the Emergency Department: A Quasi-Experimental Study

**DOI:** 10.3390/jcm15124810

**Published:** 2026-06-21

**Authors:** Laura Carbajo Martín, Ignacio Párraga-Martínez, Luis Matías Beltrán-Romero, Máximo Bernabeu Wittel, Northern Huelva Health Management Area Research Group

**Affiliations:** 1 Emergency Department, Northern Huelva Health Management Area, 21660 Minas de Riotinto, Spain; 2 Emergency and Continuous Care Group of the Spanish Society of Family and Community Medicine, 08009 Barcelona, Spain; 3 Spanish Red Cross Nursing School, University of Seville, 41009 Sevilla, Spain; 4 Health Center Zone VIII Albacete, Integrated Care Management Albacete, 02006 Albacete, Spain; iparragam@gmail.com; 5 Preventive Medicine and Public and Community Health, Faculty of Medicine of Albacete, University of Albacete, 02008 Albacete, Spain; 6 Castilla-La Mancha Health Research Institute (IDISCAM), 02071 Albacete, Spain; 7 Internal Medicine Department, Virgen del Rocío University Hospital, 41013 Sevilla, Spain; luism.beltranromero@gmail.com (L.M.B.-R.); mbernabeu@us.es (M.B.W.); 8 Department of Medicine, University of Seville, 41004 Sevilla, Spain

**Keywords:** point-of-care ultrasound, emergency department, abdominal pain, quality of care, patient satisfaction, POCUS

## Abstract

**Objectives:** To evaluate the impact of Point-of-Care Ultrasound (POCUS) performed by family physicians on the management of abdominal pain in the emergency department, assessing its effect on length of stay, performance of complementary diagnostic tests, diagnostic concordance, and patient satisfaction. **Methods:** Quasi-experimental pilot study with a control group conducted in a hospital emergency department. A total of 222 adult patients with abdominal pain were included and allocated according to the attending professional (with or without ultrasound training). Clinical, care-related, and patient-satisfaction variables (SERVPERF questionnaire) were analyzed. Non-parametric statistical tests were used, and multiple linear regression analyses were performed. **Results:** The POCUS group showed a shorter length of stay (3.46 vs. 4.41 h; *p* = 0.022) and a lower number of plain radiographies (16.8% vs. 69.9%; *p* < 0.001) and CT scans (*p* = 0.034). Diagnostic concordance was significantly higher in the experimental group (99.2% vs. 75.7%; *p* < 0.001). Overall satisfaction with received care was also higher in the intervention group (*p* < 0.001), with significant differences observed across all evaluated dimensions. The multivariate model explained 26.6% of the variability, with patient satisfaction emerging as a positive predictor. **Conclusions:** POCUS improves the quality of care in emergency departments by reducing length of stay and the use of complementary diagnostic tests while increasing diagnostic accuracy and patient satisfaction. Its implementation can be considered an effective and potentially cost-effective strategy; however, further studies with greater methodological robustness are required to validate the development of standardized composite indexes.

## 1. Introduction

Ultrasound is a procedure that uses high-energy ultrasound waves to visualize different tissues. While being a harmless technique for patients that complements the physical examination, it requires the acquisition of an adequate learning curve in order to correctly interpret images or manage artifacts [1]. The use of ultrasound began to become widespread in the field of obstetrics [2], and later on the acronym FAST (Focused Assessment with Sonography for Trauma) was coined in the emergency setting [3]. Subsequently, as a result of the widespread adoption of this bedside technique by professionals from a wide range of disciplines, the term Point-of-Care Ultrasound (POCUS) emerged [4]. The use of POCUS has been implemented in emergency departments, both in trauma patients and other conditions, such as shock of undifferentiated origin. The use of POCUS could potentially reduce healthcare costs [5].

Abdominal pain is a very common reason for presentation to emergency departments. A review of these cases conducted in 2010 indicated that non-specific abdominal pain (24.0–44.0%) constitutes the largest proportion of underlying pathologies, followed by acute appendicitis (15.9–28.1%), acute biliary disease (2.9–9.7%), and intestinal obstruction or diverticulitis in older adults [6].

The diagnostic utility of POCUS appears to be a particularly relevant tool when applied to specific clinical scenarios in the context of acute abdominal pain, rather than indiscriminately. The available evidence supports its use in suspected acute biliary disease, renal colic/hydronephrosis, abdominal aortic aneurysm, intestinal obstruction, appendicitis, and detection of free fluid in selected clinical settings. The diagnostic accuracy of ultrasound has been assessed in the literature, showing a high correlation between biliary emergency findings reported by emergency physicians and those reported by radiologists [7]. Other reviews have also shown that ultrasound performed by emergency department physicians has high specificity and moderate sensitivity for the diagnosis of acute cholecystitis [8]. In addition, studies on nephro-urological conditions have shown that moderate or severe hydronephrosis detected by clinical ultrasound suggests the presence of larger calculi [9]. Therefore, the use of POCUS could potentially modify the management of specific patient groups that may benefit from it [10].

Many patients presenting to the emergency department with abdominal pain require various complementary diagnostic tests, such as plain radiography. However, the overuse of these tests leads to unnecessary radiation exposure and increases waiting times in emergency services [11].

Finally, in the evaluation of the patient’s experience of their care process, various questionnaires are available, such as the SERVPERF (see Table A1), which explores several dimensions related to healthcare quality (tangibles, reliability, responsiveness, assurance, and empathy). This instrument has been employed in different settings, including outpatient services [12], hospital departments [13], Primary Care [14], as well as emergency departments, where the questionnaire has been validated [15].

Despite the growing evidence supporting the utility of POCUS in emergency departments, data on its overall impact on quality of care—encompassing clinical variables, effectiveness, and patient satisfaction in an integrated manner—remain limited. This study hypothesized that POCUS may assist emergency department professionals in ruling out serious causes of abdominal pain, as well as identifying other pathologies suitable for outpatient follow-up. Therefore, the present research aimed to analyze the effect of the systematic implementation of POCUS examination in the care of patients presenting with abdominal pain in the emergency setting, as a complementary measure to usual practice.

## 2. Materials and Methods

A quasi-experimental pilot study on feasibility and viability with a control group was designed and conducted in the Emergency Department of Hospital de Riotinto (Minas de Riotinto, Huelva, Spain), a public regional hospital within the Northern Huelva Health Management Division. As part of the Spanish Health System, the hospital care provision is publicly funded and universal, including emergency care, and patients may access the emergency department either directly or following referral from primary care or out-of-hospital emergency services. Inclusion criteria were patients over 18 years of age presenting to the emergency department with new-onset abdominal pain and the capacity to understand the study and provide written informed consent. Exclusion criteria were defined to ensure patient safety, obtaining valid informed consent, and adequate follow-up. Thus, patients were excluded if they presented with a life-threatening emergency requiring immediate care, such as triage priority 1, or any condition preventing them from understanding the study information and providing voluntary informed consent. Other exclusion criteria were severe cognitive impairment, unresolved language barriers, pregnancy, morbid obesity limiting adequate ultrasound examination, severe psychiatric disease, hospital admission within the previous three months, or expected follow-up outside the public health system. Patients attended by professionals trained in the performance of POCUS were allocated to the experimental group, contrary to those attended by professionals without ultrasound training who were assigned to the control group. Family physicians in the intervention group received specific theoretical and practical training in abdominal POCUS before participating in the study. The training focused on the evaluation of acute abdominal pain, including hepatobiliary, urinary tract and free fluid, as well as identification of findings requiring confirmatory radiological imaging or urgent referral. A consensus session with the Radiology Department was also conducted to standardize the scanning approach, interpretation, reporting, and referral criteria. No external certification process was required.

The sample size was calculated based on estimates from previous studies, using. length of stay as the primary outcome. The intervention effect was defined as a 25% reduction in the mean length of stay in the experimental group compared with the control group. A former trial [16] reported the average length of stay of patients with non-traumatic abdominal pain in the emergency department to be 5.2 ± 3 h. To detect a 25% reduction (1.3 h) in the intervention group, assuming a significance level of 0.05 and a statistical power of 80%, a minimum of 84 patients per group was required based on Student’s *t*-test for independent samples. Considering a dropout rate of 15%, the number of patients to be recruited was 194 (97 per group). Finally, the study sample comprised a total of 222 patients. All participants provided written informed consent after an appropriate explanation of the study. The study was approved by the Huelva Ethics Committee for Clinical Research and was conducted in accordance with the Declaration of Helsinki.

The Emergency Department of Riotinto Hospital was strategically chosen for the selection of participants. The study sample could not be randomized. Due to the care dynamics and service organization, the allocation of participants depended on the clinician responsible for their care at the time of consultation. Therefore, whether that professional was trained in the use of POCUS or not determined if the patient was assigned to the experimental or control group, respectively. Additionally, there is an ethical consideration that, once a family physician acquires the technical training and skills necessary to perform POCUS, it is not acceptable to deny patients access to an intervention they can potentially benefit from.

With respect to the intervention, upon admission to the emergency department, each patient was assigned to a study group based on the professional who attended them. POCUS was performed only on patients in the intervention group using a Versana Premier ultrasound system (GE HealthCare, Chicago, IL, USA). When clinically indicated, a physician from the radiology department performed a conventional ultrasound examination on patients in both groups. When POCUS findings were clinically relevant, the need for conventional ultrasound was determined based on the urgency of the suspected condition and its potential impact on immediate management. In cases indicative of acute cholecystitis or other conditions requiring urgent treatment, hospital admission, or urgent specialist assessment, a conventional ultrasound examination performed by a radiologist was requested before making definitive management decisions. This reflects routine practice in the emergency department, as the POCUS device is not connected to the hospital imaging network, which prevents these images from being uploaded in the hospital data system, unlike radiology-performed ultrasound that provides a formal report and imaging register in the electronic medical record. Conversely, when POCUS identified a deferrable finding, such as uncomplicated gallstones not eligible for admission, an urgent radiology ultrasound was not requested. In such cases, patients were referred for outpatient specialist consultation, scheduling conventional ultrasound prior to that consultation to guide subsequent definitive management.

The electronic medical records of all patients included in the study were subsequently reviewed to analyze the evolution of the care process initiated in the emergency department. The first follow-up review was conducted one month after the emergency visit, and the second at three months. Only if the clinical process had not been resolved at that point was the follow-up period extended for an additional six months to confirm clinical outcomes.

The main variable was the performance of POCUS. Other recorded outcomes included ultrasound findings, length of stay, other complementary diagnostic tests performed, subsequent visits, as well as sociodemographic variables (age, sex, residence municipality, distance to the hospital), clinical variables (health conditions, medication use, physical examination parameters), source of referral, discharge or referral destination, and final diagnosis.

The Ultrasound Care Quality Index (UCQI) was developed ad hoc for this exploratory study, based on the objectives and the main outcome variables defined in the protocol. The components contributing to the index were selected to reflect clinically relevant dimensions of emergency care in patients with acute abdominal pain, including length of stay, use of complementary imaging tests, diagnostic concordance, and patient-perceived quality of care. Thus, the UCQI was conceived as a composite measure to provide an overall assessment of the potential impact of POCUS on the emergency care process. This index was generated from the following study variables: length of stay in the emergency department, request for plain radiography, request for conventional ultrasound, and diagnostic agreement. All these were treated as dichotomous variables, including length of stay, which was previously recoded as dichotomous using the median as the cutoff point (values above the median were coded as 0 and values below as 1).

Descriptive analyses were conducted using proportions and appropriate measures of central tendency and dispersion. Quantitative variables were expressed by their mean (standard deviation) and qualitative variables through percentages. The normality of quantitative variables was evaluated using the Shapiro–Wilk test. When the assumption of normality was not met, between-group comparisons were conducted using non-parametric methods, specifically the Mann–Whitney *U*-test or Spearman’s rank correlation coefficient.

For the assessment of length of stay in the emergency department, this variable was firstly recoded based on the median length of stay (0 = below the median, 1 = above the median) and compared using the chi-squared test. The use of plain radiography was also analyzed using the chi-squared test. Referrals and patient characteristics were examined using non-parametric tests such as the Mann–Whitney *U*- or Kruskal–Wallis tests. Spearman’s correlation coefficient was used to explore the relationship between waiting time and overall satisfaction.

Between-group comparisons were performed using the Mann–Whitney *U*-test, and the association with overall satisfaction was assessed using Spearman’s rank correlation coefficient. Independent predictors of the UCQI were identified through multiple linear regression analysis using a stepwise approach. Firstly, assumptions of linearity, normality of residuals, and absence of multicollinearity of the included variables were verified. Results are expressed as non-standardized coefficients (B) with their corresponding 95% confidence intervals (95% CI), along with the coefficient of determination (R^2^) and adjusted R^2^ as measures of model fit. All statistical analyses were performed using IBM SPSS Statistics for Windows, version 25.0 (IBM Corp., Armonk, NY, USA). Statistical significance was set at *p* < 0.05.

## 3. Results

The intervention was conducted between March 2023 and June 2024, with a total sample of 222 patients, of whom 36.5% were men and 63.5% were women. One hundred and three patients were allocated to the control group and 119 to the experimental group. The median age was 54.5 years [interquartile range (IQR) = 33].

The median distance from the patients’ place of residence to the hospital was 31 km (IQR = 36). The mean length of stay in the emergency department was 3 h 44 min (95% CI: 3 h 27 min–4 h 02 min), with a standard deviation (SD) of 2 h 11 min, and the median length of stay was 3 h 12 min (IQR = 1 h 44 min). The baseline characteristics of both study groups are shown in Table 1.

In terms of ultrasound findings, the most frequent corresponded to renal conditions (13.1%), followed by gallbladder disorders (9.5%). Table 2 shows the distribution of the most frequent POCUS findings in the experimental group, stratified by sex.

Final diagnoses were coded according to the ICD-10 classification during the clinical follow-up. The most frequent final diagnoses were non-specific abdominal pain (33.8%), renal colic (18.5%), constipation (6.8%), gallstones (6.8%), epigastric pain (6.3%), urinary tract infection (5.4%), gastroenteritis (4.1%), and acute pyelonephritis (3.6%). Acute abdominal conditions of clinical relevance were also identified, including diverticulitis (2.3%), acute cholecystitis (2.3%), acute pancreatitis (1.8%), pelvic inflammatory disease (1.4%), appendicitis (0.9%), intestinal obstruction (0.5%), and ascites (0.5%).

Length of stay was converted into decimal format to facilitate analysis and interpretation. The mean length of stay in the experimental group was 3.46 h (SD 3.35), with a median of 3.12 h (IQR 2.36), which were greater than in the control group, with a mean stay of 4.41 h (SD 1.45) and a median of 3.52 h (IQR 2.36). To analyze length of stay and determine the existence of between-group differences, the variable was further recoded into a dichotomous format using the median value as the cutoff. The length of stay was shorter for patients who underwent POCUS, a difference that attained statistical significance (*p* = 0.022).

In the control group, the proportion of patients requiring plain radiography was significantly higher than in the experimental group [72/103 (69.9%) vs. 20/119 (16.8%), with statistically significant differences observed (*p* < 0.001). In addition, a higher number of CT scans were performed in this control group, with the result also reaching statistical significance (χ^2^ = 4.493, df = 1, *p* = 0.034). No differences were observed in the need for conventional ultrasound performed at the radiology service (12 and 13 cases in the control and experimental groups, respectively). However, when analyzing whether POCUS revealed any findings or not, only one patient without findings in POCUS performed by a family physician underwent conventional ultrasound. This result was statistically significant (χ^2^ = 11.402, df = 1, *p* = 0.001). These outcomes are shown in Table 3.

Of the patients included in the study, only six ultimately required surgical intervention: in the control group, two patients were diagnosed with appendicitis and two with cholecystitis, while in the experimental group, two more patients were diagnosed with cholecystitis.

To analyze diagnostic accuracy, follow-ups of all participants’ electronic medical records were performed at one and three months after the emergency visit. Final diagnosis could be confirmed in 196 (88.29%) patients. Diagnostic agreement reached 99.2% in the experimental group (only one case was not confirmed), whereas the concordance rate in the control group was 75.7% (78 of 103 patients). This relationship was statistically significant (χ^2^ = 29.32, df = 1, *p* < 0.001). Cohen’s Kappa coefficient was calculated to assess agreement between POCUS findings and the final diagnosis, demonstrating weak agreement (Κ = 0.247; *p* < 0.001).

The overall patient satisfaction measured with the SERVPERF questionnaire showed a median score of 123 (IQR = 19) and a mean of 118.97 (SD 14.17) points, ranging from a minimum of 76 to a maximum of 133 points. In the experimental group, the mean satisfaction score was 124.51 points (SD 9.33) with a median of 126 points (IQR 14), whereas in the control group the mean was 112.57 points (SD 16.02) with a median of 114 points (IQR 33). Satisfaction levels were compared between the experimental and control groups using the Mann–Whitney *U*-test, revealing statistically significant between-group differences (Mann–Whitney *U* = 3456.5, Z = −5.642, *p* < 0.001), with higher satisfaction in the group where POCUS was performed compared to the control (mean rank 133.95 vs. 85.56 points, respectively). Table 4 shows overall satisfaction data.

The persistence of statistical significance was assessed across factors, revealing statistically significant between-group differences across all the domains of the questionnaire: Reliability and Assurance (Mann–Whitney *U* = 3440.00; *p* < 0.001), Accessibility and Responsiveness (Mann–Whitney *U* = 3345.00; *p* < 0.001), and Tangible Elements (Mann–Whitney *U* = 4147.50; *p* < 0.001). In all cases, mean ranks were higher in the intervention group in which family physicians performed POCUS. Detailed data can be found in Table 5.

The relationship between waiting time and overall satisfaction was evaluated using Spearman’s rank correlation coefficient, revealing a weak but statistically significant negative correlation (ρ = −0.146, *p* = 0.029). No relationships were found between overall satisfaction and other parameters such as age, comorbidity, medication use, or other clinical variables. However, a statistically significant association was observed between overall satisfaction and diagnostic agreement, with mean ranks of 115.12 versus 84.21 points (Mann–Whitney *U* = 1838.500, Z = −2.323, *p* = 0.020) in the experimental and control groups, respectively.

The UCQI was compared between the control and experimental groups using the Mann–Whitney *U*-test. Higher mean ranks were observed in the POCUS group with statistically significant between-group differences (mean ranks 137.40 vs. 81.57 points in the experimental and control groups, respectively; Mann–Whitney *U* = 3046.000; Z = −6.757; *p* < 0.001). The relationship between care quality using POCUS, as measured by this index, and overall patient satisfaction was explored using Spearman’s correlation coefficient. The results showed a positive and statistically significant association between the two variables, although of low magnitude (ρ = 0.285; *p* < 0.001; *n* = 222).

A multiple linear regression analysis was conducted to identify factors that could affect the UCQI. Firstly, various combinations of variables were explored using a stepwise regression procedure to identify the model that best explained the variability of the index. After testing multiple options, the final model included the following variables: overall satisfaction, referral for hospital admission, referral to a different hospital specialty, subsequent visit to the emergency department, heart rate, and pain level measured using a visual analog scale (VAS). The best-fit model reached statistical significance and explained 26.6% of the total variability of the UCQI (R^2^ = 0.266; adjusted R^2^ = 0.244; *p* < 0.001). Overall patient satisfaction showed a positive and significant association with the UCQI (B = 0.005; *p* < 0.001), whereas hospital admission (B = −0.201; *p* = 0.001), subsequent visits to the emergency department (B = −0.120; p = 0.002), heart rate (B = −0.002; *p* = 0.031), and VAS pain (B = −0.013; *p* = 0.044) were negatively associated with perceived quality of care. In contrast, referral to another medical specialty was positively associated with the index (B = 0.148; *p* < 0.001). Finally, the absence of collinearity among predictor variables was verified. All results are shown in Table 6.

## 4. Discussion

A systematic review in 2019 stated that POCUS may contribute to the optimization of healthcare costs [5]. The results of the present research showed that the use of POCUS in patients presenting to the emergency department with abdominal pain reduced both length of stay and the performance of other complementary diagnostic tests, in addition to improving the patient’s satisfaction with the care provided.

Patients in the control group stayed in the emergency department for a significantly longer period than those in the experimental group, similarly to reports in previous studies. A retrospective study analyzing whether performing POCUS within the first hour after admission in non-traumatic abdominal pain was associated with patient flow, use of healthcare resources, or quality of care concluded that early ultrasound was linked to a shorter hospital stay without compromising patient safety [17]. Another former study showed that early performance of POCUS in patients with acute flank pain was associated with a shorter hospital stay without increasing morbidity or mortality [18]. Therefore, the use of POCUS by the clinician providing initial care in an emergency department may be considered an optimization strategy and could be proposed in the near future for cost-effectiveness purposes.

With regard to the characteristics of care in this specific emergency department, the highest number of visits for abdominal pain occurred on the first day of the week, Monday, followed by Thursday, while Sunday was the day with the lowest attendance. These data are consistent with those of numerous studies reporting higher emergency department attendance on Mondays and lower attendance during weekends [19]. The observed patterns are not limited to a specific geographic setting, as studies conducted in urban hospitals show the same predominance of emergency visits on Mondays compared with other days of the week [20]. Emergency department overcrowding represents a major challenge in healthcare management. Given that emergency departments receive the highest influx of patients on Mondays, strategies for the reinforcement or optimization of patient inflow–outflow should be strengthened on these days. However, the variability is substantial, and further studies are warranted to better characterize severity in these patients. The present study did not find an association between patient condition severity and weekday attendance to the emergency department, indicating that the phenomenon represents an increase in volume rather than complexity [21].

On the other hand, participants in the experimental group underwent fewer plain radiographs and CT scans than those in the control group. Plain radiography was included as a care-process variable as it is frequently used in patients with abdominal pain and represents a potentially modifiable diagnostic resource. In our setting, it is primarily requested when intestinal obstruction, constipation, nonspecific abdominal pain, or other abdominal conditions are suspected. However, its use may vary according to local practice and clinician judgment. The methodological characteristics of this study limit direct comparison of these results, as the available evidence on the reduction in complementary tests among patients with abdominal pain is scarce. However, studies have shown that the use of lung POCUS in critically ill patients reduces the performance of radiographs and CT scans in intensive care units [22]. POCUS has also been found to lower the average number of differential diagnoses, which in turn helps reduce the number of complementary tests [23]. Nevertheless, the low CT scan utilization rate observed in our cohort should be interpreted with caution, as it likely reflects the study context, patient selection, and local diagnostic pathways rather than the effect of POCUS alone. The rate of patients discharged home was also lower in the experimental group, where POCUS was performed, contrary to referrals for consultation with other specialties, which were more frequent in the experimental group. Although this may be partly attributable to between-group differences in the severity of abdominal conditions, it may also be explained by greater diagnostic accuracy and the need for more comprehensive evaluation based on POCUS findings. In addition, previous research has demonstrated the potential of POCUS to improve patient referral processes [24].

Subsequent emergency visits for the same reason accounted for up to 36.9% of the study sample. Abdominal pain is a very common complaint among patients presenting to emergency departments, and revisiting rates reported in the literature are highly variable. Some interesting strategies involve scheduling reassessment visits within the following hours to examine the occurrence of clinically relevant changes or alternative diagnoses [25]. Although the control group showed a higher number of subsequent visits for the same reason, no between-group differences were found in the attendance setting (primary care vs. hospital emergency).

Diagnostic agreement at the one- and three-month follow-ups was achieved in 88.29% of the overall sample (196 patients). This is slightly higher than figures reported in a former retrospective study (81.99%), although this included only hospitalized patients [26]. The analysis of diagnostic agreement at emergency department discharge revealed that the concordance rate in the experimental group was higher than in the control group, a difference that reached statistical significance. This finding suggests that POCUS reduces diagnostic uncertainty by allowing a more precise patient assessment. The utility of performing POCUS in the diagnosis and management of non-traumatic acute abdominal pain has been established in clinical guidelines and protocols, given its effectiveness for achieving accurate diagnosis with a high concordance level [27].

The evaluation of healthcare quality has been approached from multiple perspectives. The SERVQUAL and SERVPERF questionnaires are part of the instruments developed in recent years to assess perceived quality, with the latter being particularly relevant. A 2019 study pointed out SERVPERF as the most appropriate instrument for healthcare services [28]. The reliability and validity of the SERVPERF questionnaire are consistent with other studies that have employed it to measure satisfaction and quality in various healthcare settings [29].

To the best of our knowledge, there are no similar studies in our country assessing perceived care quality among patients undergoing POCUS performed by family physicians. This hinders the comparison of outcomes with similar healthcare models. Nevertheless, the use of validated quality questionnaires has also been explored in previous research [30]. Some studies have already incorporated the patient’s perception, reporting significantly higher satisfaction scores when bedside ultrasound was used [31]. Higher levels of patient satisfaction may also be explained by a “positive care” effect, similar to a placebo effect, which provides socio-emotional benefits in receptive patients, as suggested by another study reporting an association between higher levels of satisfaction and improvements in care efficiency [32]. Furthermore, the widespread availability of portable ultrasound devices also entailed an overall greater patient satisfaction [33]. However, none of the referenced studies employed a validated instrument, such as the one used for this research.

This study observed that the implementation of POCUS in the emergency department results in a significant improvement in quality of care, as evaluated through the composite index UCQI, which was designed ad hoc. Other investigations that assessed these outcomes separately concluded that POCUS improves diagnostic accuracy, reduces care provision times and length of stay in emergency departments, and even improves care among patients in need for subsequent visits for the same reason [34]. This multidimensional approach is also consistent with studies using Delphi techniques in hospital emergency departments, which assessed key dimensions such as care provision times, triage, and total length of stay [35].

## 5. Conclusions

In conclusion, the use of POCUS in the emergency department practice for patients with abdominal pain reduces length of stay and the performance of complementary diagnostic tests, increases diagnostic agreement, and improves the care provision as experienced by the patient.

## 6. Limitations

The study design, including a control group, allowed assessing the impact of using POCUS compared with usual management, while enabling a multidimensional analysis that incorporated clinical variables, care provision times, diagnostic agreement, and patient satisfaction. These strengths offer a comprehensive overview of the quality of care in patients managed with and without POCUS.

Although the UCQI was useful as an exploratory composite measure in this study, it has not yet undergone formal validation. Therefore, a new research line has been initiated to develop and validate a quality index for emergency care, incorporating indicators related to efficiency, diagnostic appropriateness, resource use, and patient-perceived quality.

On the other hand, the sample size was not sufficient for performing secondary analyses by subgroups, which limits statistical power. Another limitation is the potential variability in experience among health professionals, which could have affected diagnostic accuracy or examination times. However, the standardized training provided may have contributed to homogenizing practice and reducing this variability. The length of stay depends on multiple variables, such as workload or the need for additional tests, which may have acted as confounding factors; however, the inclusion of a control group for outcome comparison likely helped mitigate this bias. In terms of subsequent visits to the emergency department, detailed information was not available about the underlying reasons, which would be of interest to differentiate between expected relapses, accessibility issues, or other circumstances. Finally, the study was not designed to compare the diagnostic accuracy of POCUS versus other imaging modalities. Although complementary tests and final diagnoses at follow-up visits were recorded, no specific analysis was performed to link individual POCUS findings with subsequent conventional ultrasound or CT results. Future studies should evaluate this correlation to better identify the clinical scenarios in which POCUS is sufficient as an initial imaging tool and those requiring additional imaging.

## Figures and Tables

**Table 1:** Descriptive analysis of the study sample by groups at the baseline.

Variables	Control Group *n* = 103 (%)	Experimental Group *n* = 119 (%)	Total *n* = 222 (%)	*p*-Value
Age				0.005 *
18–30 years	25 (24.3)	9 (7.6)	34 (15.3)	
31–50 years	28 (27.2)	36 (30.3)	64 (28.8)	
51–65 years	23 (22.3)	29 (24.4)	52 (23.4)	
>65 years	27 (26.2)	45 (37.8)	72 (32.4)	
Referral from				
Primary Care	25 (24.3)	27 (22.7)	52 (23.4)	0.781
Own’s initiative	61 (59.2)	68 (57.1)	129 (58.1)	0.754
Another medical specialty	1 (1.0)	0 (0)	1 (0.5)	0.281
Primary Care Emergencies	16 (15.5)	24 (20.2)	40 (18.0)	0.370
Referral to				
Home Discharge	92 (89.3)	77 (64.7)	169 (76.1)	<0.001 *
Consultation with another Specialty	2 (1.9)	34 (28.6)	36 (16.2)	<0.001 *
Observation	0 (0)	1 (0.8)	1 (0.5)	0.351
Hospital admission	9 (8.7)	7 (5.9)	16 (7.2)	0.412
Subsequent visits	44 (42.7)	35 (29.4)	79 (35.6)	0.039 *
To Primary Care	24 (23.3)	17 (14.3)	41 (18.5)	0.084
To Primary Care Emergencies	8 (7.8)	11 (9.2)	19 (8.6)	0.695
To Hospital Emergency department	25 (24.3)	22 (18.5)	47 (21.2)	0.293
To other care settings	1 (1.0)	0 (0)	1 (0.5)	0.281

* Statistically significant difference (*p* < 0.05).

**Table 2:** POCUS findings by sex.

Findings	Men (*n* = 81) (%)	Women (*n* = 141) (%)	Total (*n* = 222) (%)	*p*-Value
Renal pelvis dilation	7 (8.6)	9 (6.4)	16 (7.2)	0.090
Gallstones	8 (9.9)	7 (5.0)	15 (6.8)	
Renal cyst	7 (8.6)	6 (4.3)	13 (5.9)	
Bile sludge	4 (4.9)	2 (1.4)	6 (2.7)	
Other *	2 (2.5)	3 (2.1)	5 (2.3)	
Hepatic steatosis	1 (1.2)	3 (2.1)	4 (1.8)	
Hepatic cyst	3 (3.7)	0 (0)	3 (1.4)	
Hepatic angioma	2 (2.5)	0 (0)	2 (0.9)	
Cholecystitis	1 (1.2)	1 (0.7)	2 (0.9)	
Cirrhotic liver	0 (0)	1 (0.7)	1 (0.5)	

* Renal agenesis, cholecystectomy, corpus luteum, renal angiolipoma, bladder polyp, ovarian cyst.

**Table 3:** Relationship between the performance of POCUS by family physicians and complementary tests (plain RX, CT scan, traditional ultrasound).

Tests	Plain RX (%)	*p*	CT Scan (%)	*p*	Conventional Ultrasound (%)	*p*
No POCUS performed by family physician	72 (78.3)	<0.001	6 (85.7)	0.034	12 (48.0)	0.864
POCUS performed by family physician	20 (21.7)		1 (14.3)		13 (52.0)	
Conventional ultrasound without findings	15 (75.0)	0.041	1 (100)	0.357	1 (8.3)	0.001
Conventional ultrasound with findings	5 (25.0)		0 (0)		11 (91.7)	

**Table 4:** Between-group comparison of patients’ satisfaction level measured with the SERVPERF questionnaire (see Table A1). *p*-value was obtained using the Mann–Whitney *U*-test as the distribution did not fit normality.

	Control Group				Experimental Group				
Item	Minimum	Maximum	Mean	SD	Minimum	Maximum	Mean	SD	*p*-Value
1	1	7	5.86	1.085	1	7	6.21	0.982	0.006
2	3	7	5.83	0.981	3	7	6.43	0.776	>0.001
3	3	7	5.49	1.145	1	7	6.01	1.305	>0.001
4	1	7	5.73	1.113	2	7	6.37	0.973	>0.001
5	4	7	5.97	0.902	2	7	6.69	0.710	>0.001
6	2	7	5.92	1.118	4	7	6.62	0.689	>0.001
7	3	7	5.96	0.979	4	7	6.56	0.721	>0.001
8	4	7	6.09	0.898	3	7	6.68	0.650	>0.001
9	4	7	6.12	0.932	5	7	6.69	0.517	>0.001
10	4	7	6.19	0.908	5	7	6.81	0.412	>0.001
11	4	7	6.16	0.916	4	7	6.74	0.589	>0.001
12	4	7	5.98	0.896	4	7	6.63	0.636	>0.001
13	4	7	6.13	0.860	4	7	6.70	0.561	>0.001
14	4	7	6.01	0.857	5	7	6.62	0.611	>0.001
15	4	7	6.07	0.910	4	7	6.73	0.563	>0.001
16	4	7	5.93	0.973	4	7	6.64	0.563	>0.001
17	2	7	5.64	1.212	4	7	6.48	0.779	>0.001
18	2	7	5.76	1.124	3	7	6.46	0.779	>0.001
19	2	7	5.74	1.146	2	7	6.50	0.812	>0.001

**Table 5:** Satisfaction level by SERVPERF dimension and study group.

Dimension	Group	Average Range	Range Addition	Mann–Whitney *U*	Z*	*p*-Value
Reliability and Assurance	Control	85.40	8796.00	3440	−5.793	<0.001
	Experimental	134.09	15,957.00			
Accessibility and Empathy	Control	84.48	8701.00	3345	−5.971	<0.001
	Experimental	134.89	16,052.00			
Tangible Elements	Control	92.27	9503.50	4147.5	−4.218	<0.001
	Experimental	128.15	15,249.50			

Z* refers to the standardized test statistic for the Mann–Whitney *U*-test.

**Table 6:** Results of the multiple linear regression model for the ultrasound care quality index (UCQI).

Variable	B	Standardized β	95% CI	t	*p*-Value
Constant	0.370	–	0.055–0.684	2.319	0.021
Overall satisfaction	0.005	0.283	0.003–0.007	4.592	<0.001
Referral for hospital admission	−0.201	−0.216	−0.316–−0.086	−3.453	0.001
Referral to another specialty consultation	0.148	0.224	0.066–0.230	3.560	<0.001
Subsequent visit to the hospital emergency department	−0.120	−0.202	−0.194–−0.046	−3.208	0.002
Heart rate	−0.002	−0.136	−0.004–0.000	−2.168	0.031
VAS (perceived pain)	−0.013	−0.128	−0.025–0.000	−2.031	0.044

Note: B, unstandardized regression coefficient; β, standardized regression coefficient; CI, confidence interval; t, *t*-test statistic.

## Data Availability

The datasets generated or analyzed during the current study are available from the corresponding author on reasonable request. The datasets will be available from the corresponding author upon reasonable request.

## Appendix A

**Table A1:** Satisfaction questionnaire adapted from the SERVPERF. Likert scale (1–7) from least to most satisfied.

Items	Strongly Disagree					Strongly Agree	
Physical facilities are adequate for care provision	1	2	3	4	5	6	7
2. The emergency department has up-to-date equipment	1	2	3	4	5	6	7
3. The emergency department has sufficient capacity to attend to the population	1	2	3	4	5	6	7
4. If the patient needs to resolve any questions or concerns, they are attended to within a reasonable time.	1	2	3	4	5	6	7
5. Employees provides efficient and high-quality care provision	1	2	3	4	5	6	7
6. The patient was attended to quickly and efficiently	1	2	3	4	5	6	7
7. Provides services as promised	1	2	3	4	5	6	7
8. Employees show sincere interest in solving patient problems	1	2	3	4	5	6	7
9. Performs the service right the first time	1	2	3	4	5	6	7
10. Employees show willingness to help whenever required	1	2	3	4	5	6	7
11. Employee behavior inspires confidence	1	2	3	4	5	6	7
12. Employees have adequate knowledge to resolve doubts accurately	1	2	3	4	5	6	7
13. Employees have adequate knowledge to answer questions	1	2	3	4	5	6	7
14. Care provision responds to the patient’s needs	1	2	3	4	5	6	7
15. Employees care about the patient’s interests and needs	1	2	3	4	5	6	7
16. The emergency department addresses patients’ health needs	1	2	3	4	5	6	7
17. Waiting time to be attended was adequate	1	2	3	4	5	6	7
18. Waiting time for test results was adequate	1	2	3	4	5	6	7
19. Overall waiting time was adequate	1	2	3	4	5	6	7
